# Histomorphometrical Analyses of the Mouse Suprachiasmatic Nucleus

**DOI:** 10.21769/BioProtoc.5704

**Published:** 2026-06-05

**Authors:** Sophia A.M.B. Villanueva, Frank Y. Lee, Olivia N. Hitchcock, Esteban C. Dell’Angelica, Christopher S. Colwell, Cristina A. Ghiani

**Affiliations:** 1 Department of Psychiatry & Biobehavioral Sciences, Semel Institute for Neuroscience and Human Behavior, Institute for Developmental Disabilities Research Center, David Geffen School of Medicine, University of California, Los Angeles, Los Angeles, CA, USA; 2 Department of Human Genetics, David Geffen School of Medicine, University of California, Los Angeles, Los Angeles, CA, USA; 3 Department of Pathology and Laboratory Medicine, David Geffen School of Medicine, University of California, Los Angeles, Los Angeles, CA, USA

**Keywords:** Suprachiasmatic nucleus, Circadian rhythms, Retino-hypothalamic tract, Retinal–SCN innervation, Cholera toxin, Plot profile, Animal models

## Abstract

The mammalian central circadian clock resides in the suprachiasmatic nucleus (SCN) of the hypothalamus in the brain and is responsible for coordinating daily rhythms of biological processes spanning from gene expression to behavior. Light, the primary environmental zeitgeber, entrains the SCN via melanopsin-expressing intrinsically photosensitive retinal ganglion cells that project through the retino-hypothalamic tract. Altered circadian rhythms are common in individuals diagnosed with neurodevelopmental and neurodegenerative disorders, and often, associated with structural alterations of the SCN and impaired retinal input; importantly, these anomalies can be recapitulated in animal models. Here, we describe step-by-step protocols for quantitative histomorphometrical analysis of the SCN and the assessment of retinal–SCN connectivity, previously used in mouse models of neurodevelopmental and neurodegenerative disorders. These include measurement of the SCN area, perimeter, height and width using Nissl- or DAPI-stained coronal sections, as well as densitometric and plot profile analyses of cholera toxin β-subunit–labeled retinal projections using Axiovision or Fiji/ImageJ. The protocols incorporate standardized region-of-interest, measurements by masked observers, and consistent scaling procedures to enhance reproducibility. These methods provide a rigorous framework for detecting structural anomalies and connectivity defects in the circadian system and can be broadly applied to other experimental models of circadian dysfunction.

Key features

• Histomorphometrical analyses of the SCN can provide anatomical bases to understand altered sleep and circadian rhythms in animal models of disease.

• Exploration of retinal–SCN connectivity to facilitate the identification of the underlying causes of deficits in the response to photic cues in animal models of disease.

• The protocols described here employ widely used and accessible software and provide rigorous but easy-to-follow instructions.

• These analyses do not require expensive staining procedures and can be easily implemented in any laboratory.

• Strengths for reproducibility: usage of fixed region-of-interest (ROI), measurements averaged from multiple sections per animal, masked observers thoroughly trained.

## Graphical overview


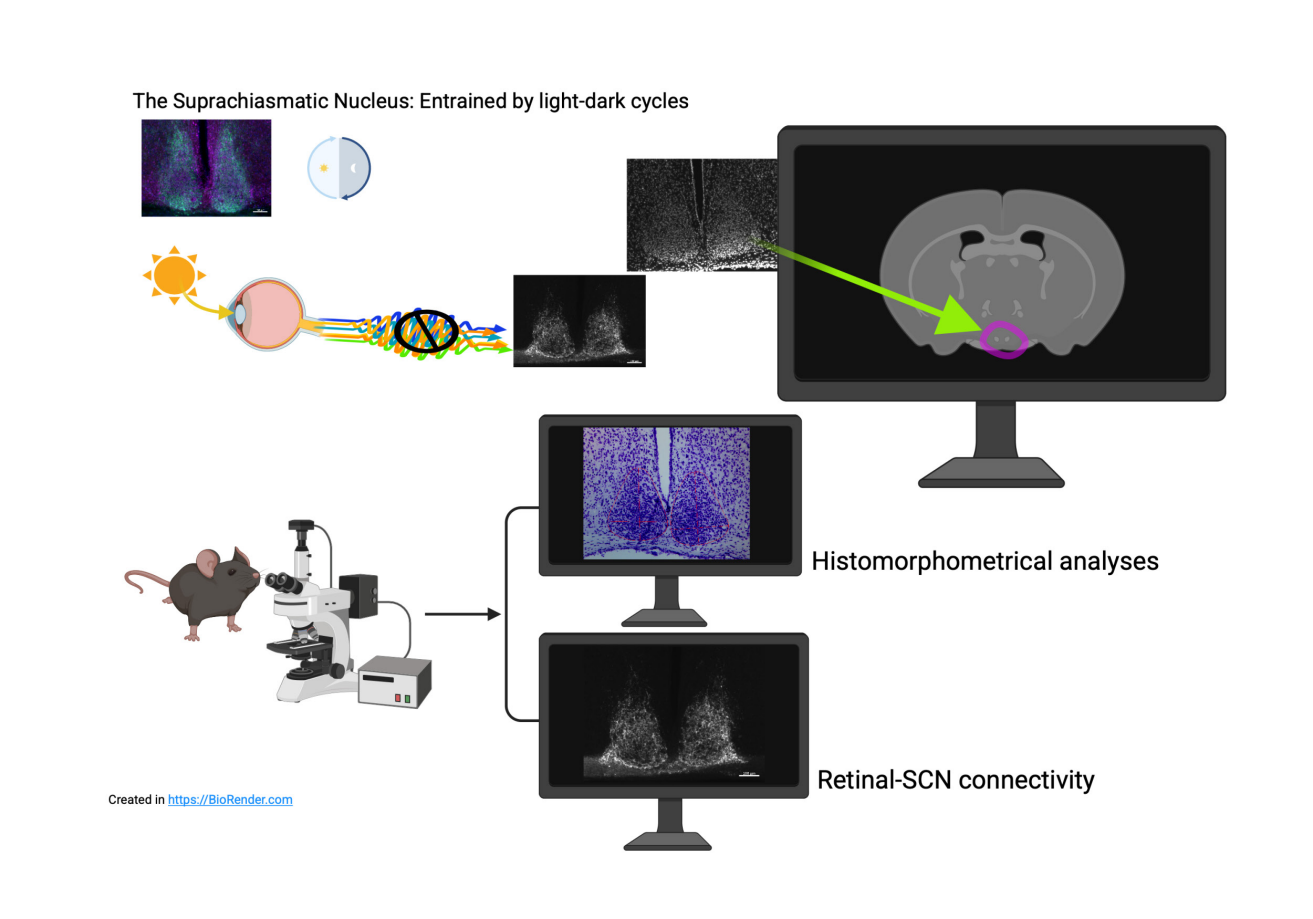


**Workflow for the histomorphometrical and connectivity analyses in the mouse suprachiasmatic nucleus (SCN)**

## Background

**Figure 1.**
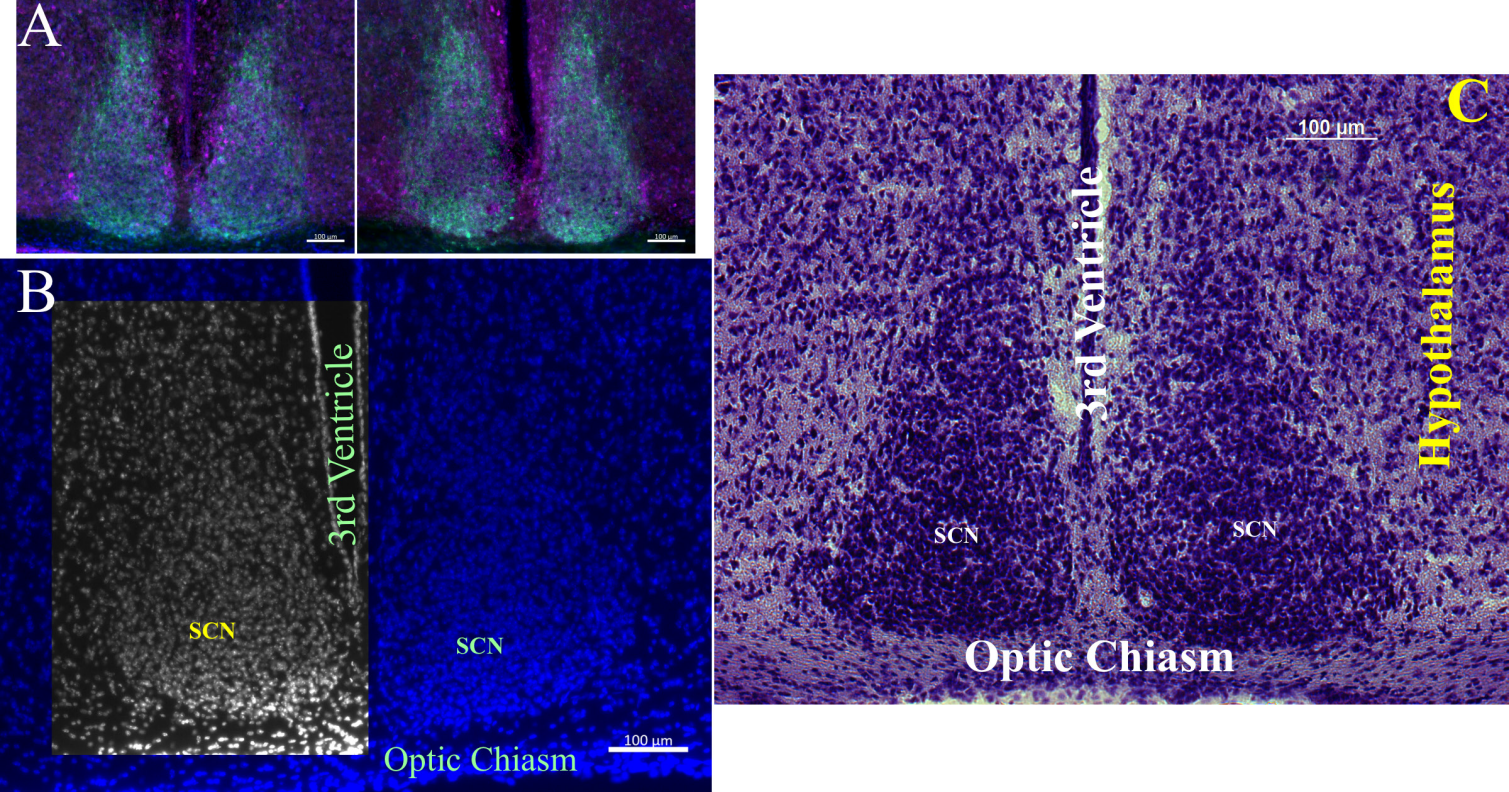
Representative images of the suprachiasmatic nucleus (SCN) in mouse coronal brain sections. (A) Immunofluorescent staining of two of the main neuronal cell types present in the SCN: vasoactive-intestinal peptide (VIP)-positive cells (green) are identifiable in the ventrolateral part of the SCN with long processes projecting into the dorsal part; arginine vasopressin (AVP)-positive cells (magenta) are present in the dorsomedial part of the SCN. (B) DAPI- or (C) Nissl-stained coronal sections containing the SCN. In these images, the cell density characteristic of the SCN can be seen in comparison to the rest of the anterior hypothalamus. The location of the third ventricle and of the optic chiasm is shown in relation to the SCN. Note that all the images shown in this and subsequent figures were acquired using a 10× objective and the scale bars are equal to 100 μm.

All living organisms (from unicellular to humans) possess intrinsic biological clocks, which, in mammals, are orchestrated and harmonized by the master clock, the suprachiasmatic nucleus (SCN) in the anterior hypothalamus of the brain [1,2]. This bilateral structure, positioned above the optic chiasm flanking the third ventricle, contains approximately 20,000 densely interconnected neurons (Figure 1) that exhibit endogenous spontaneous oscillations with a period of around (*circa*) 24 hours [3]. The central clock undergoes daily resynchronization dictated by external cues (zeitgebers), with environmental light being the primary entraining signal and the fast/fed cycle a secondary one; the SCN, then, maintains the synchrony of peripheral clocks throughout the central nervous system and body’s organs via both neural and humoral output pathways [4,5].

Alterations in circadian rhythms can be highly detrimental to physiological and cognitive functions in both humans and other animal species. In modern society, circadian disruptions have become increasingly prevalent and, regrettably, may severely impact children and adolescents during sensitive windows of brain development [6]. A growing body of evidence links circadian misalignment and sleep disruptions/deprivation to a range of medical conditions; for example, shift workers not only experience impairments of cognitive function and performance in everyday life but are also at higher risk of developing mood and neurological disorders, cardiometabolic syndromes as well as some forms of cancer [7]. Furthermore, individuals with neurodegenerative or neurodevelopmental disorders frequently present with disturbances in their sleep/wake cycles, including difficulties initiating and maintaining sleep [8,9].

Several of the circadian and sleep-related symptoms in affected individuals have been recapitulated in preclinical animal models [10–13]. Notwithstanding these functional disturbances being accompanied by structural anomalies of the SCN and deficits in retinal innervation [14–19], standardized and reproducible quantitative approaches to assess anatomical changes and/or faulty connectivity in the diseased circadian system still remain limited.

Preclinical models have provided critical insight into alterations of the SCN, the molecular clockwork and the retino-hypothalamic tract (RHT) [14–19]; with the latter constituting the primary conduit through which photic information is conveyed from the retina to the SCN. Light is detected in the retina, at least in part, by melanopsin-expressing intrinsically photosensitive retinal ganglionic cells (ipRGCs), the axons of which synapse on SCN neurons and other targets in the hypothalamus [20–22]. Impaired responsiveness of the SCN to photic cues can hinder its daily synchronization and circadian entrainment (or re-entrainment if needed), compromising the resetting and proper alignment of downstream clocks disseminated in the rest of the central nervous system and body. Over time, such misalignment can have detrimental effects on people’s health, wellness and well-being, and in the context of neurodevelopmental and neurodegenerative disorders, exacerbate cognitive, cardiometabolic, behavioral and physiological symptoms [7,9,22,23].

Sleep disturbances are common among individuals affected by Fragile X syndrome (FXS), a neurodevelopmental disorder caused by the abnormal expansion of CGG repeats in the *FMR1* gene [24]. Recently, we have reported that the *Fmr1* knockout (KO) mouse, a model of FXS, exhibits deficits in the circadian behavioral response to light, for instance delayed re-entrainment following shifts in the light-dark cycle and reduced light-induced phase shifts of the activity rhythms [19]. These mice also display aberrant social and repetitive behaviors, the severity of which correlates with sleep duration and fragmentation. Using the histomorphometric and connectivity analyses described here, we identified structural abnormalities in the SCN and deficits in the RHT that likely contribute to the observed impaired photic entrainment. Aided by these findings, we implemented a scheduled feeding regimen (6-h feeding/18-h fasting) as a non-photic circadian-based intervention to repristinate the rhythms in the mutants independently of light cues. This regimen greatly improved the rhythms in activity, increased social interactions, and reduced repetitive behaviors in the mutants.

Circadian rhythms are driven by a molecular feedback loop characterized by ~24-h oscillations in the expression of core clock proteins [4,5]. In an earlier study [16], using these analytical approaches, we reported the altered expression of the core component of the molecular circadian clock Period 2 (Per2) in another model of neurodevelopmental psychiatric disorders, namely the Biogenesis of Lysosome-related Organelles Complex-1 (BLOC-1)-deficient mice. Persistently high levels of Per2 expression were identified in both the SCN and the hippocampus of BLOC-1-deficient mice, suggesting a malfunctioning molecular clock potentially contributing to the observed circadian dysfunction and associated behavioral aberrations. In addition, these mutants exhibited an enlarged SCN. Similar structural alterations have been identified in mouse models of Rett Syndrome [14] and Huntington’s Disease [15], but not in the *Cntnap2* KO mouse, a model of autism spectrum disorders [25], further supporting the utility of quantitative morphometric analyses in detecting disease-related changes in the central circadian pacemaker. Noticeably, these protocols have proven useful to reveal defects also in other brain areas [26,27].

Here, we provide a detailed step-by-step workflow for the quantification of SCN morphometry performed using either Nissl- or DAPI-stained coronal mouse brain sections and the assessment of the retinal–SCN connectivity using the Cholera Toxin β-subunit neurotracing in coronal mouse brain sections. These protocols should be useful tools for identifying anomalous morphology and deficits in the input and output pathways of the SCN, and perhaps, potential targets for novel circadian (and non-) therapeutic strategies.

## Materials and reagents

**Reagents**

1. Cholera toxin subunit B (Recombinant), Alexa Fluor^TM^ 555 conjugate (Invitrogen^TM^, catalog number: C34776)

## Equipment

1. Zeiss Axioskop 50 equipped with color Axiocam 208

2. Objective Plan-APOCHROMAT 10×/0.32 (Zeiss, catalog number: 4440630)

3. Objective Plan-APOCHROMAT 10× (Zeiss, catalog number: 420340-9901)

4. Zeiss AxioImager M2 microscope equipped with a motorized stage, an AxioCam MRm and the ApoTome imaging system (any comparable system can be used)

## Software and datasets

1. AxioVision software, Zeiss version 4.5, Carl Zeiss AG, Germany,

https://www.micro-shop.zeiss.com/en/us/system/software-axiovision+software-products/1007/

2. NIH ImageJ software with the Fiji image processing package, https://imagej.net

3. Microsoft^®^ Excel for Mac (Version 16.106.1)

4. GraphPad Prism version 10.0.0 for Mac, GraphPad Software, www.graphpad.com

5. Zen software, Carl Zeiss AG, https://www.zeiss.com/microscopy/en/products/software/zeiss-zen.html

6. Zen Lite software, Carl Zeiss AG, https://www.zeiss.com/microscopy/us/products/software/zeiss-zen-lite.html

*Note: A free copy of Zen Lite can be attained by creating a MyZEISS account to download the ZEISS Microscopy Installer using the link above. This is not just a viewer for the CZI files but it allows performing image acquisition, processing and analysis.*

**General notes**

The SCN is an oblong structure, with three anatomically identifiable subdivisions: anterior, middle, and posterior, based on both shape and functional connectivity. In most of our work, we focused on the largest subdivision, the mid-SCN (Figure 1) but have also taken measurements spanning from the most anterior to the most posterior extent of the nucleus. To obtain and combine these measurements from multiple mouse brains, it is important to identify the (five to seven) sections that contain the largest cross-sectional area of the nucleus (i.e., the mid-SCN); hence, we typically stain between 10 and 15 consecutive sections paired on the rostral (anterior) to caudal (posterior) axis per animal for a total of 5–6 mice per experimental group.

Accurate delineation of the SCN borders presents methodological challenges due to the gradual transitions in cell density between the SCN and the surrounding hypothalamic region (Figure 1B, C; Supplementary Figure 1). The left and right mid-SCN are identified in Nissl- or DAPI-stained coronal brain sections by location, based on the morphology of the third ventricle and the optic chiasm, as well as cell density (Figure 1B, C). Because the borders of the DAPI- or Nissl-defined SCN are not sharply demarcated and somewhat arbitrary, measurements are performed independently by two observers masked to the experimental groups. Inter-observer consistency can be verified by having two or more observers score the same material independently and quantifying how closely their measurements agree; their training should continue until the intraclass correlation coefficient (ICC) approaches values equal or greater than 0.75. We note that the SCN lacks morphological features that would allow unambiguous and objective identification of its borders; accordingly, successful implementation of these protocols relies on proper training, extensive practice and experience (see Supplementary Figure 1 for some examples of outlined SCN). In a like manner, quantification of retinal afferent fiber density and spatial distribution requires consistent region-of-interest (ROI) selection and standardized image analysis parameters. Variability in image acquisition, scaling, and analysis workflows can significantly affect morphometric and densitometric measurements.

## Procedure

The procedures for the injections of the cholera toxin, brain harvesting, tissue processing and Nissl staining can be found in the supplementary methods file as well as in prior publications included in the validation section [14–16,19].

The protocols below provide a detailed step-by-step workflow to: 1) obtain measures of the area, perimeter, height and width of the SCN identified by either Nissl or DAPI staining in consecutive mouse coronal brain sections (20 or 50 μm thickness, respectively; Figure 1B, C) [14–16,19]; 2) analyze the light input to the SCN via the retino-hypothalamic pathway using the neurotracer cholera toxin (β-subunit) conjugated to Alexa Fluor^TM^ 555 [19] in consecutive coronal brain sections (50 μm thickness).

**A. Image acquisition of the middle SCN**

1. Acquire images (2D) of the sections containing the SCN (from rostral to caudal). We use the Zen Zeiss software and a Zeiss AxioImager M2 microscope equipped with a motorized stage and an AxioCam MRm for fluorescently stained sections or a Zeiss Axioskop equipped with a color Axiocam if Nissl-stained; comparable imaging systems can be used.

2. In the *Acquisition mode* tab of Zen, activate the channels for all the fluorophores [Cyanine3 (Cy3) for the cholera toxin and DAPI in our case]. The SCN is identified by location, shape and cell density in the ventral (inferior) part of the coronal brain sections using a 10× objective (Figure 1) to include both left and right SCN in one image.

3. In the same tab, select *Live*, focus and determine the exposure time for all the channels by clicking on *set exposure* for the sections of each animal in all the experimental groups included in a set; then, choose the lowest value and keep it constant while imaging all the sections of the animals in that set. Determine the exposure time for the second set, the third and so on until the images for all the sets are acquired. See in the note below what we consider to be “a set”.

4. Capture images of the left and right SCN from all the sections, since it will be easier to identify and select the images with the largest nuclei by opening them side by side. Save the files as “*name*.czi.”

**Table 1.** Parameters used for image acquisition

	Exposure time	Gain		Bit depth	
Cholera toxin Alexa 555-conjugated	Set 1: 400 ms; Set 2: 500 ms; Set 3: 300 ms		1		12
DAPI	Between 70 and 100 ms		1		12
Nissl staining	8–10 ms				42

*Notes:*

*1. “A set” is composed of one animal per experimental group or condition (wild-type control, wild-type treated, mutant control, mutant treated) and usually, we aim for at least 5 sets, i.e., the number of independent samples or biological replicates per experimental group or condition is n = 5. All brains in “a set” are usually processed (frozen, cut, and stained) together (see Supplementary Methods).*

*2. It is good practice to minimize the exposure time for fluorescent images to avoid acquiring overexposed images or bleaching the sections. The time usually does not differ much between the sets of animals (Table 1); nevertheless, we determine/measure the exposure time for each set. This is a delicate, albeit time-consuming step.*

**B. Morphometrical analyses of the SCN using Zeiss AxioVision software**

**Figure 2.**
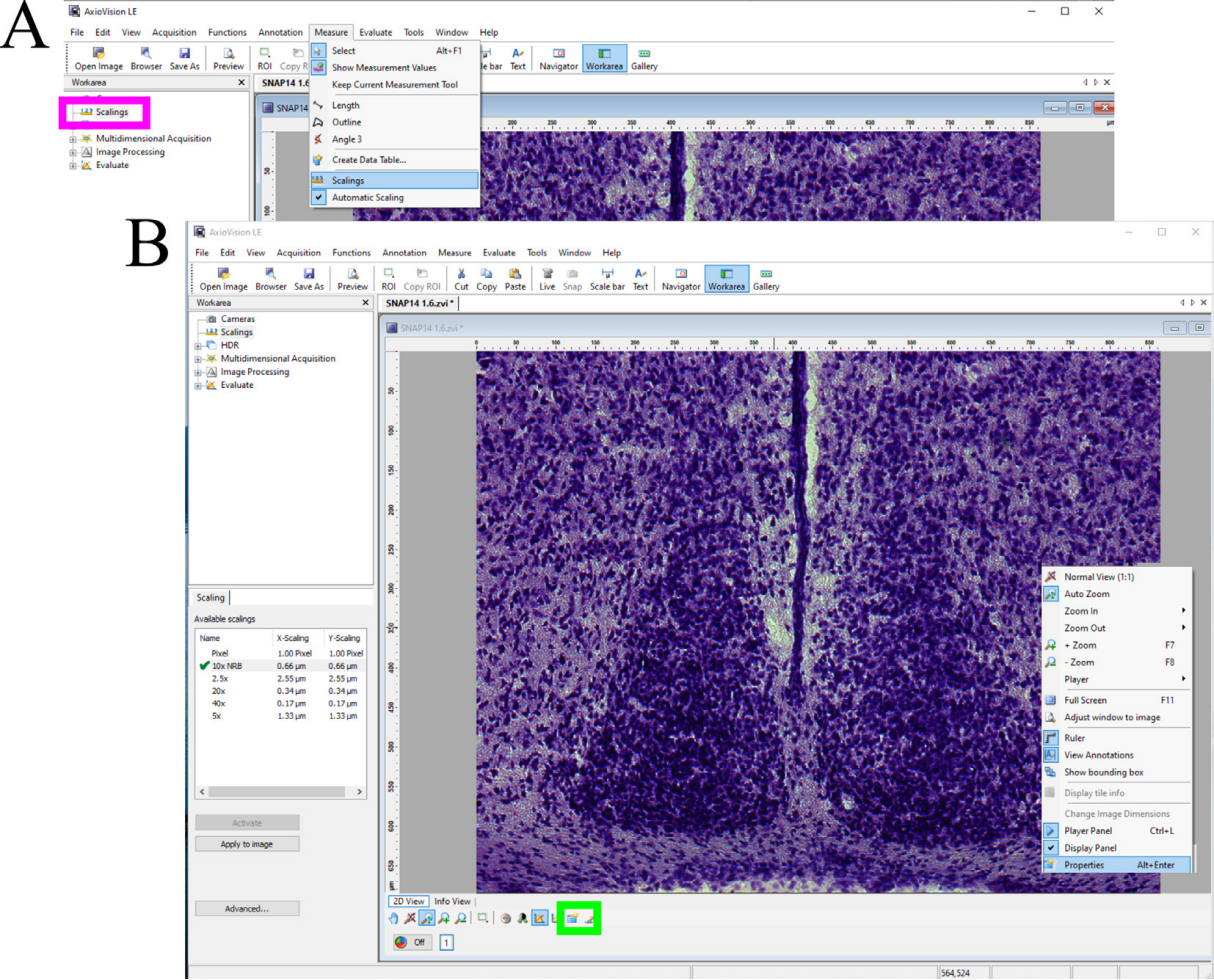
Using the Zeiss AxioVision Software for histomorphometrical analyses of the suprachiasmatic nucleus (SCN). (A) After opening the image in AxioVision, step 1 is to apply the correct scaling (magenta box). (B) The *Properties* dialog window can be opened from the menu at the bottom of the image (green box) or by right-clicking on the image.

1. To estimate the area, perimeter, height and width of the left and right SCN, convert the .*czi* files (Figure 1B, C) to .*zvi* to be opened in the AxioVision software (Zeiss). This is one of the options given by ZEN under *save as* or *export*.

2. Launch the AxioVision software and open the .*zvi* file.

3. Select and apply the correct scaling to the image, so the measurements will be in the correct (“real”) units. This can be done (Figure 2) by either opening it from the *Measure* menu (Figure 2A; it will open in a separate window) or clicking on *Scalings* (magenta box in Figure 2A) in the *Work area* menu (*ctrl W* to open the latter); the menu will open below (Figure 2B).

4. Select the proper objective, click on *Activate* and then on *Apply to Image*.

5. Open the *Properties* window. There are several ways to open this:

a. From the *View* Menu.

b. From the shortcut in the toolbar menu at the bottom of the image window (green box in Figure 2B).

c. By right-clicking on the image to open the shortcut menu (Figure 2B).

**Figure 3.**
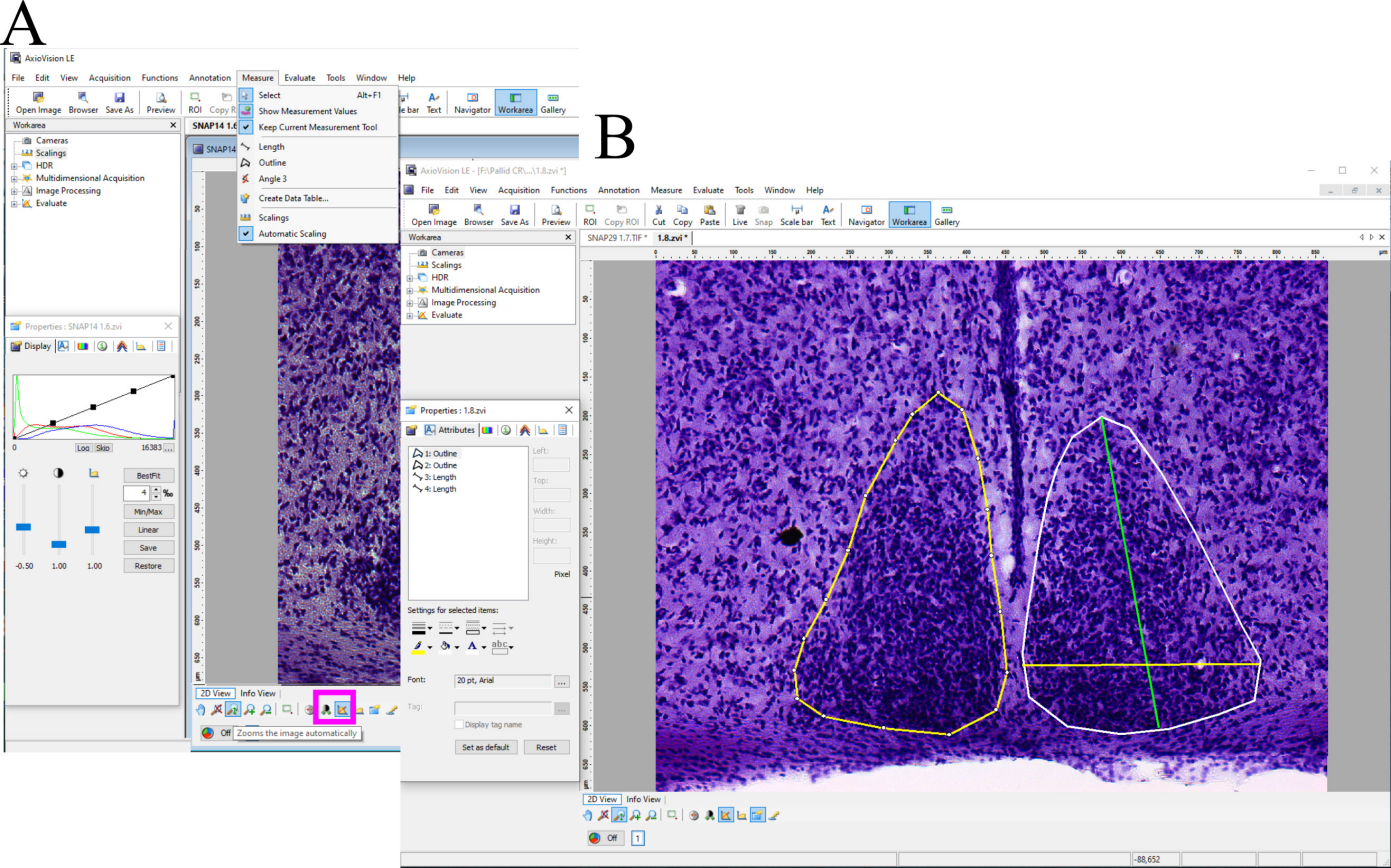
Using the Zeiss AxioVision Software for histomorphometrical analyses of the suprachiasmatic nucleus (SCN). (A) Select and apply the linear or *Min/Max* correction for an optimal display of the image from either the first tab of the *Properties* window, *Display*, or the bottom menu (magenta box). The drawing tools are found in the *Measure* menu at the top. (B) The second tab of the *Properties* window, *Attributes*, contains a series of options to modify the appearance of the drawn shapes (outlines, lines, etc.) used for the analyses. The menu at the bottom of the image contains a series of useful shortcuts, including those to resize the image.

6. In *Properties* (Figure 3A), click on the *Display* tab and select *Linear* for default display if using Nissl-stained sections or *Min/Max* for fluorescence (DAPI). Alternatively, both the linear and the *Min/Max* icons (magenta box; Figure 3A) can be found in the toolbar below the image window. These settings provide the optimal contrast and brightness to visualize the nuclei’s shape and borders, mostly based on cell density. Use the icon with the magnifier selected in the toolbar below the image (Figure 3A, B) to resize it to fit in the window. Shortcuts to zoom in and out are also present.

7. Keep the *Properties* window open; details and information about the image can be found in the other tabs.

8. Find and select the proper drawing tool in the *Measure* menu (Figure 3A). More tools can be added as needed; these can also be present as a toolbar on the top ribbon of the AxioVision window.

9. To measure the cross-sectional area and perimeter: select the *Outline or Outline spline tool* from the measure menu (Figure 3A) and draw the shape of the SCN (Figure 3B) by carefully following its border based on the difference in cell density between the nucleus and the lateral hypothalamus (see also Figure 1B, C and Supplementary Figure 1).

10. To measure the height and the width of the SCN: select and use the *Length tool* to draw a line from dorsal to ventral (or vice versa) at the tallest part (Figure 3B; green line) or from lateral to medial (or vice versa) at the widest part (Figure 3B; yellow line), respectively.

*Notes:*

*1. The lines and shapes drawn are considered* Annotations *on top of the image and can be temporarily removed by right-clicking on the image and selecting/deselecting* View Annotations *(Figure 2B, right inset).*

*2. Enlarging the image makes it difficult to see the borders of the SCN; conversely, the nucleus becomes more evident when zooming out.*

**Figure 4.**
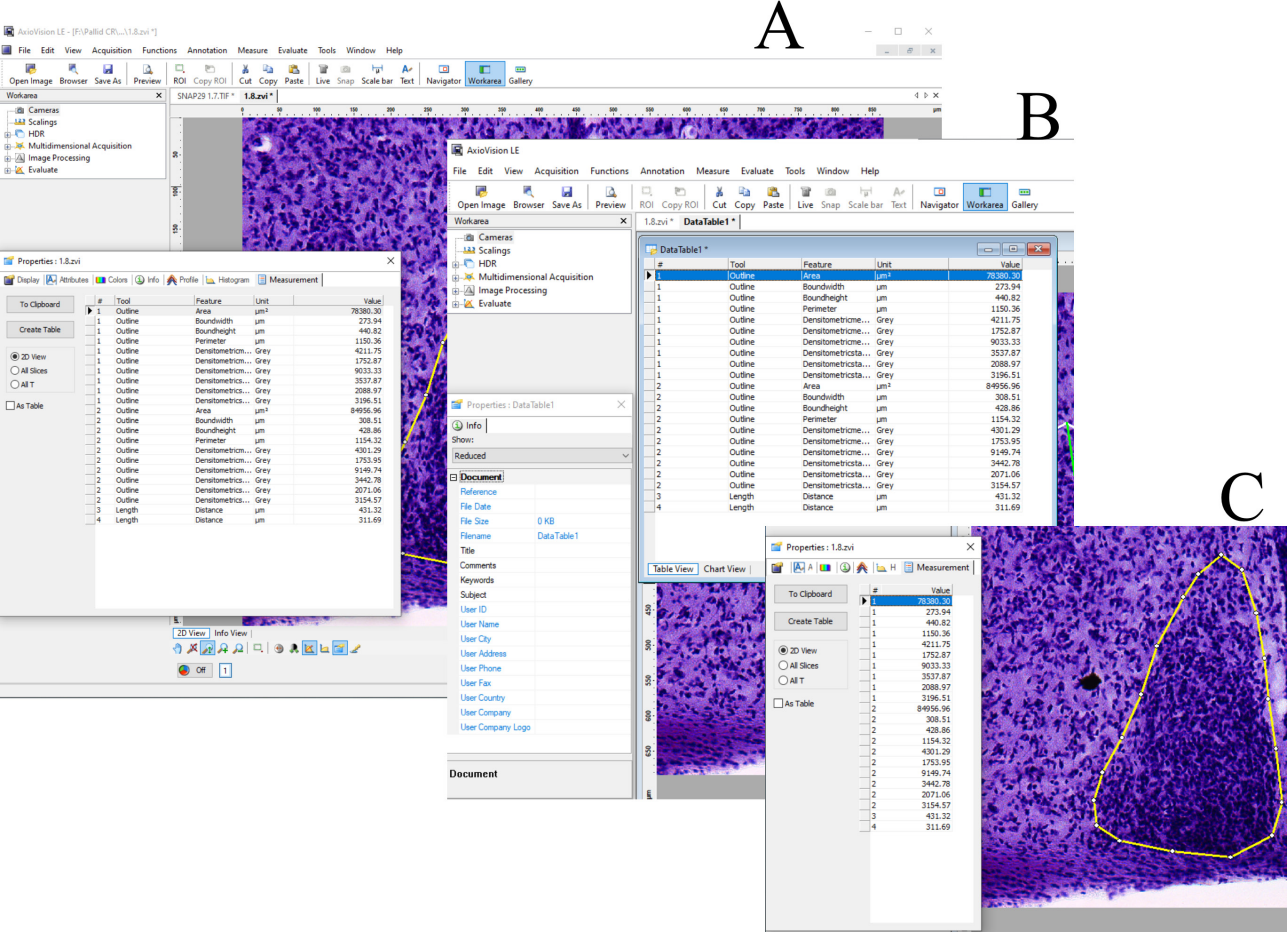
Using the Zeiss AxioVision Software for histomorphometrical analyses of the suprachiasmatic nucleus (SCN). (A) The last tab of the *Properties* window, *Measurement*, contains the values obtained. (B) These can be exported by creating a *Data Table* and saving it as .csv. (C) Clicking on a value in the *Measurement* tab highlights the associated shape/annotation in the image and vice versa.

11. In *Properties*, the *Attributes* tab (Figure 3B) contains the options to change the color, thickness, appearance or type of lines as well as the font, color and size of the text (Figure 3B). In this window, select the *Annotation* to be formatted, then choose the settings in the menus below. It is also possible to select whether to display and justify any text with the annotations.

12. Visualize the values obtained in *Measurement*, the last tab of the *Properties* window (Figure 4A), and extract them by clicking on *Create table* on the left side of this window. The table generated opens in a new window (Figure 4B); save it as *name*.csv. Alternatively, copy and paste the values needed (area, perimeter, length) into an Excel spreadsheet. These values are linked to the image, i.e., clicking on a specific annotation/shape in the image will highlight the associated value in the table and vice versa (Figure 4C).

**C. Retinal–SCN connectivity**

Anomalies or deficits in the retinal afferent fibers to the SCN can impair the ability of the organism to respond to photic cues and properly entrain and synchronize to the light cycle. The fluorescent signal of the retinal innervation of the SCN is strongest in the ventral aspect, where the retino-hypothalamic fibers reach the nuclei. Hence, we describe two methods of analysis carried out on the images of five consecutive sections per animal containing the mid-SCN: 1) analysis of the relative intensity of the cholera toxin fluorescent ipRGC processes performed separately in both the left and right whole SCN, and 2) examination of the distribution of the cholera toxin fluorescent signal for each left and right ventral SCN separately.

**Figure 5.**
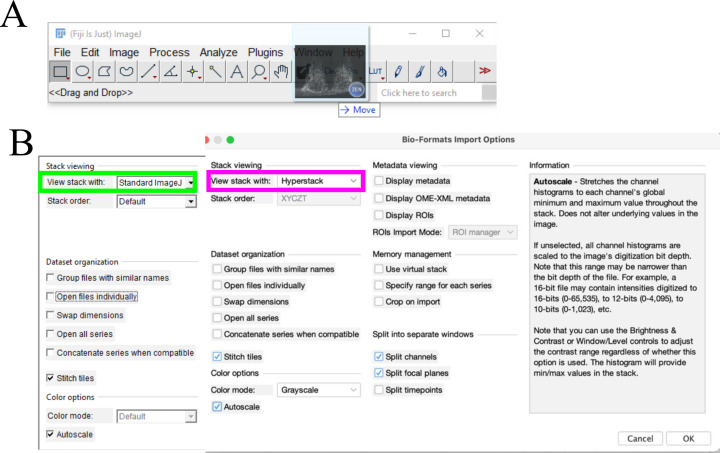
Using Fiji/ImageJ to detect deficits in the retino-hypothalamic pathway. (A) The .*czi* files of the images acquired with the Zeiss Zen software can be opened by *Drag & Drop*. (B) *Stack Viewing* window for standard images on the left (green box) and Z-Stack images on the right (magenta box). The *grayscale* option allows for optimal visualization of fluorescent images.

1. Quantification of the relative intensity of cholera toxin fluorescent ipRGC fibers in the whole SCN by scanning densitometry using the Fiji image processing package of ImageJ:

a. Launch Fiji/ImageJ.

b. Open the .*czi* file(s) by dragging and dropping into ImageJ (Figure 5A).

*Note: Do not try to open the file from the* FILE *>* OPEN *menu, as it will not work.*

c. The *drag & drop* triggers the opening of the window-menu *Bio-Formats Import Options* (Figure 5B), before the image is opened. In this menu, depending on the type of images [Z-stacks vs. single plane (2D) images] to be analyzed, check that the following are selected:

i. Single plane images: Standard image (Figure 5B, green box).

ii. Z-stack images: Hyperstack (Figure 5B, magenta box).

iii. Autoscale

iv. Color Options: Greyscale; although often the image automatically opens in black and white; if it does not, go back and check this selection.

v. Split Channels: Each channel will open in a separate window, so the DAPI image can be seen side-by-side with the cholera toxin (or another marker) image. This is very helpful to move and reposition the ROI as needed.

vi. Split Focal Planes: to be selected if the images are Z-stacks, to analyze each frame in sequence; these will open in separate windows.

vii. Click *Ok* in this and the next menu, where you are asked to choose a stack. Images will open.

*Note: These settings will also work for older versions of Fiji.*

**Figure 6.**
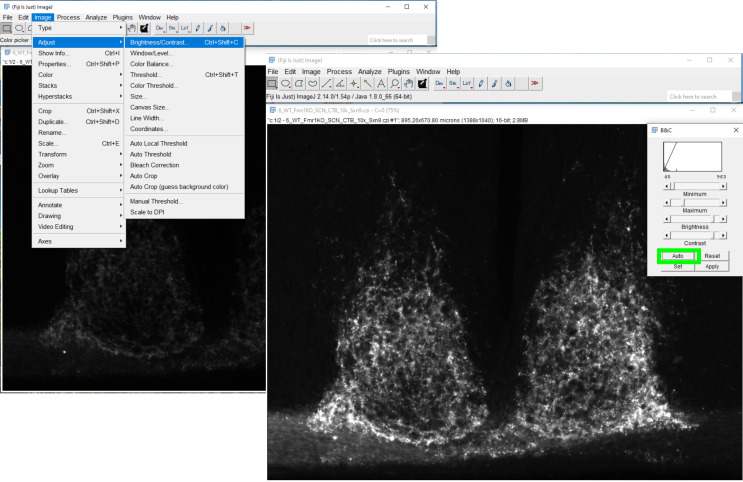
Using Fiji/ImageJ to detect deficits in the retino-hypothalamic pathway. Steps to adjust the brightness and contrast of the images, since upon opening, these are typically completely dark. Selecting *Auto* (green box) is enough to provide a good visualization of the images.

**Figure 7.**
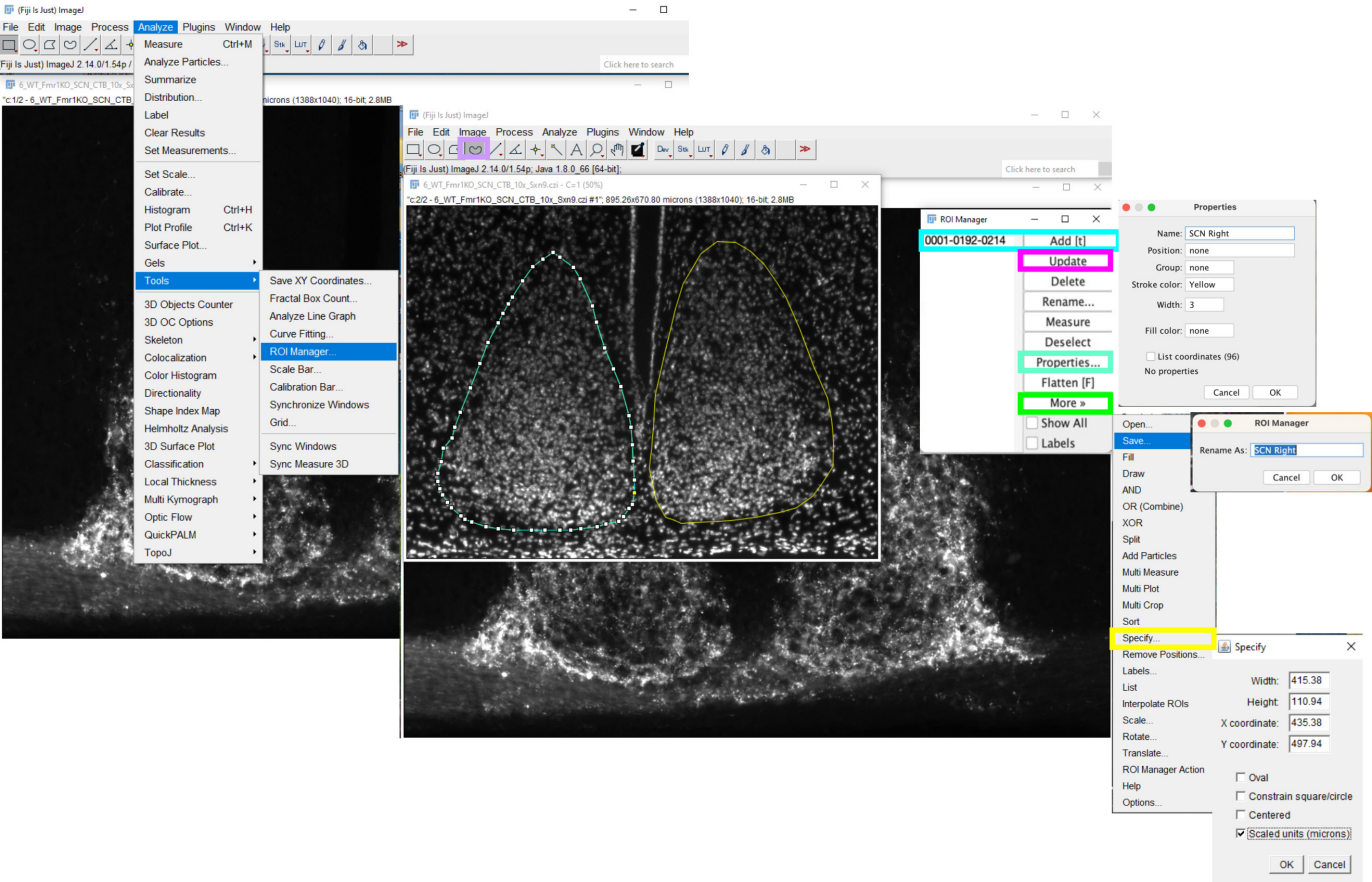
Using Fiji/ImageJ to detect deficits in the retino-hypothalamic pathway. Steps to draw the region of interest (ROI) for the left and right suprachiasmatic nucleus (SCN) using the *freehand* tool (purple box). These should be of fixed size, and great care should be taken not to inadvertently modify the size and shape if repositioned. The colored boxes indicate different commands/functions in the ROI Manager; Cyan box: click to *Add* the newly drawn ROI to the *ROI Manager*; Magenta box: *Update* to apply any change made to the ROI; Green box: *More*, opens a pop-up menu with multiple functions (open, save, specify); Turquoise box: *Properties*, to modify the appearance of the ROIs.

d. Upon opening, the images are usually quite dark, thus adjust their brightness by *Image > Adjust > Brightness/Contrast* (Figure 6). The B&C menu will open in a separate window. Select *Auto*; no other adjustment is needed (Figure 6, green box).

e. To set up one ROI for the left SCN and one for the right SCN: open the *ROI Manager* from the *Analyze menu > Tools > ROI Manager* (Figure 7). The ROI menu will open in a separate window.

f. Select the *freehand* tool (Figure 7, purple box).

g. Using the DAPI image, outline the entire left SCN (Figure 7, turquoise outline), click *ADD* in the *ROI Manager* (Figure 7, cyan box); repeat for the right SCN (Figure 7, yellow outline), and click *ADD*.

h. The ROIs can be found in the left window of the *ROI manager* (Figure 7, cyan box), rename and save in a dedicated folder by selecting *More > Save > Rename as…* (Figure 7, green box). Click on *More > Open* and navigate to the folder where the ROIs were saved to open them for subsequent sessions.

i. Before beginning the measurements, select the scale units (microns) in the *Specify* window (Figure 7, yellow box), which contains further information on the ROI, such as the size and position. Modify the color, thickness and appearance of the line of the ROIs in the *Properties* (Figure 7, turquoise box) pop-up window.

*Notes:*

*1. The left and right ROIs will be slightly different in size; still, they must be of fixed size and kept constant for all the images of the samples of the project.*

**Figure 8.**
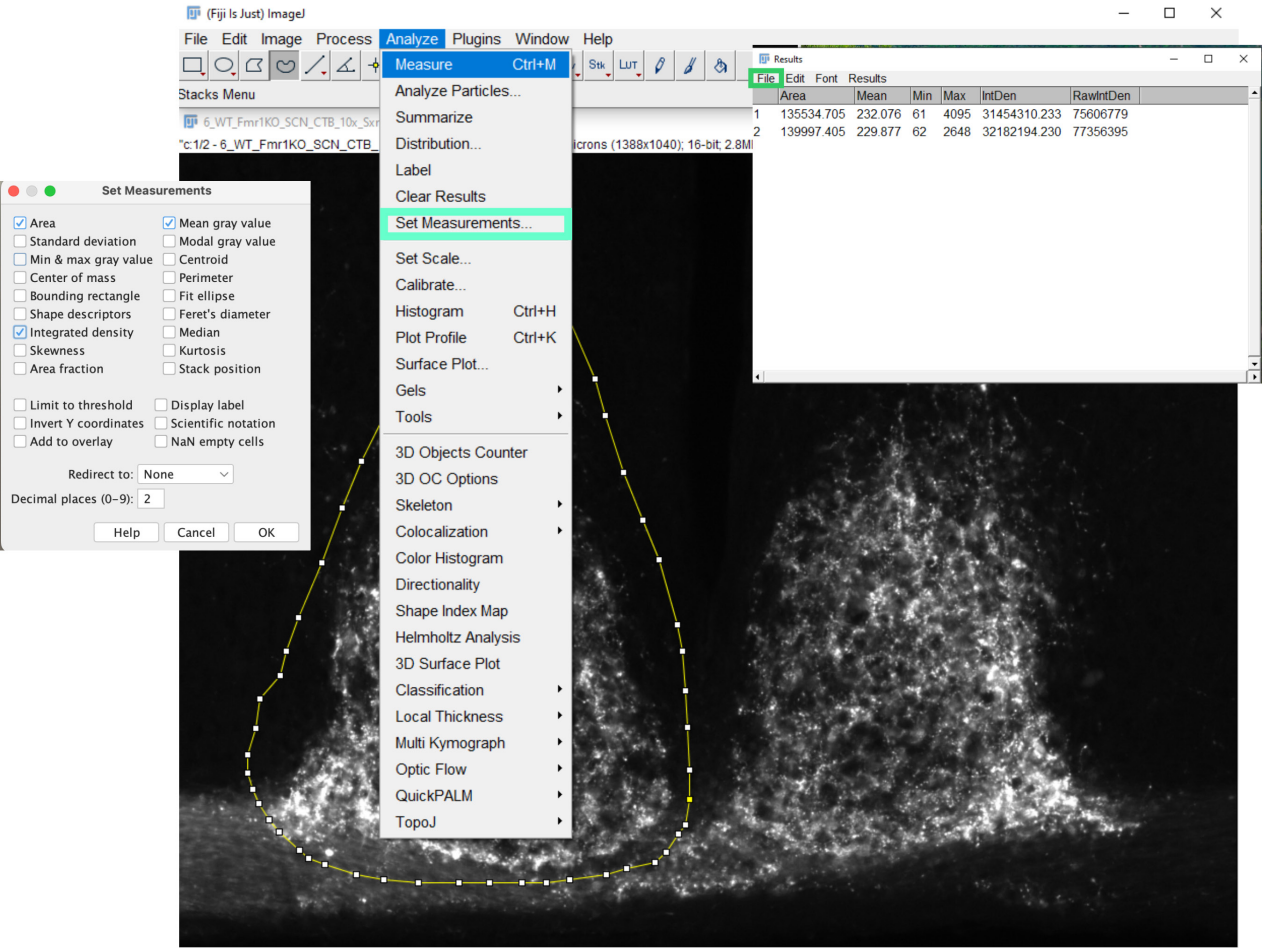
Using Fiji/ImageJ to detect deficits in the retino-hypothalamic pathway. Steps to measure by scanning densitometry the relative intensity of the cholera toxin signal in the entire suprachiasmatic nucleus (SCN). Turquoise box: *Set Measurements*, to choose the parameters to include in the analysis; Green box: *File*, to open the menu to export and save the measurements obtained.

*2. To draw the ROI, we use the DAPI image containing the largest SCN. This is identified by aligning on the screen the consecutive images of all the samples containing the central portion of the SCN and then, besides visually identifying it, by measuring those that appear with the largest cross-sectional area.*

j. To superimpose the ROI on the image of the SCN showing the cholera toxin tracer (Figure 8): select the image, then the ROI in the window of the *ROI manager*, reposition it **if** and **as** needed by using the arrows on the keyboard, to make sure that the entire SCN is within the ROI. Click on *Update* to save any changes to the position (Figure 7, magenta box). Caution should be taken to **not** change the size or shape of the ROIs when repositioning them.

*Note: It is helpful to have the cholera toxin and corresponding DAPI image open side-by-side when superimposing the ROIs, as there will be some small variations in the position of the SCN among the images due to uneven mounting and image acquisition, thus the need to reposition it from image to image. The thickness of the optic chiasm is a good landmark to limit and minimize this inconsistency when capturing the images.*

k. To add the parameters to include in the analysis, go to *Measure > Set Measurements* (Figure 8, turquoise box), and in the pop-up window, select *Mean gray value* and *Integrated density*.

l. To quantify the fluorescent signal, select *Measure on the Analyze menu* or in the *ROI manager* (Figure 7) as well as directly by *Command/Control M*. The *Results* window will open (Figure 8). Repeat for all the images to be analyzed.

m. To save the results, select *File > Save as* in the *results* window (Figure 8, green box) or right-click on this window; it will be saved as a .csv file.

*Notes:*

*1. The mean gray value gives a measure of the average brightness of the pixels within the ROI, whilst the integrated density is measured as the Mean gray values × Area of the ROI. Both are measured from at least 5 consecutive sections per animal. The values derived from the left and right SCN of each section are averaged to obtain one value per section; the values from all the sections per animal are then averaged to obtain one value per animal.*

*2. If the analysis is performed on Z-stack images, the mean gray value and integrated density are acquired from each of the most central frames (likely 10 to 15). The values obtained for each Z-stack frame of the left SCN and those for the right SCN are averaged separately to obtain one value per side; the values for the left and right SCN are averaged to obtain one value per image; all these are averaged to obtain one value per animal.*

**Figure 9.**
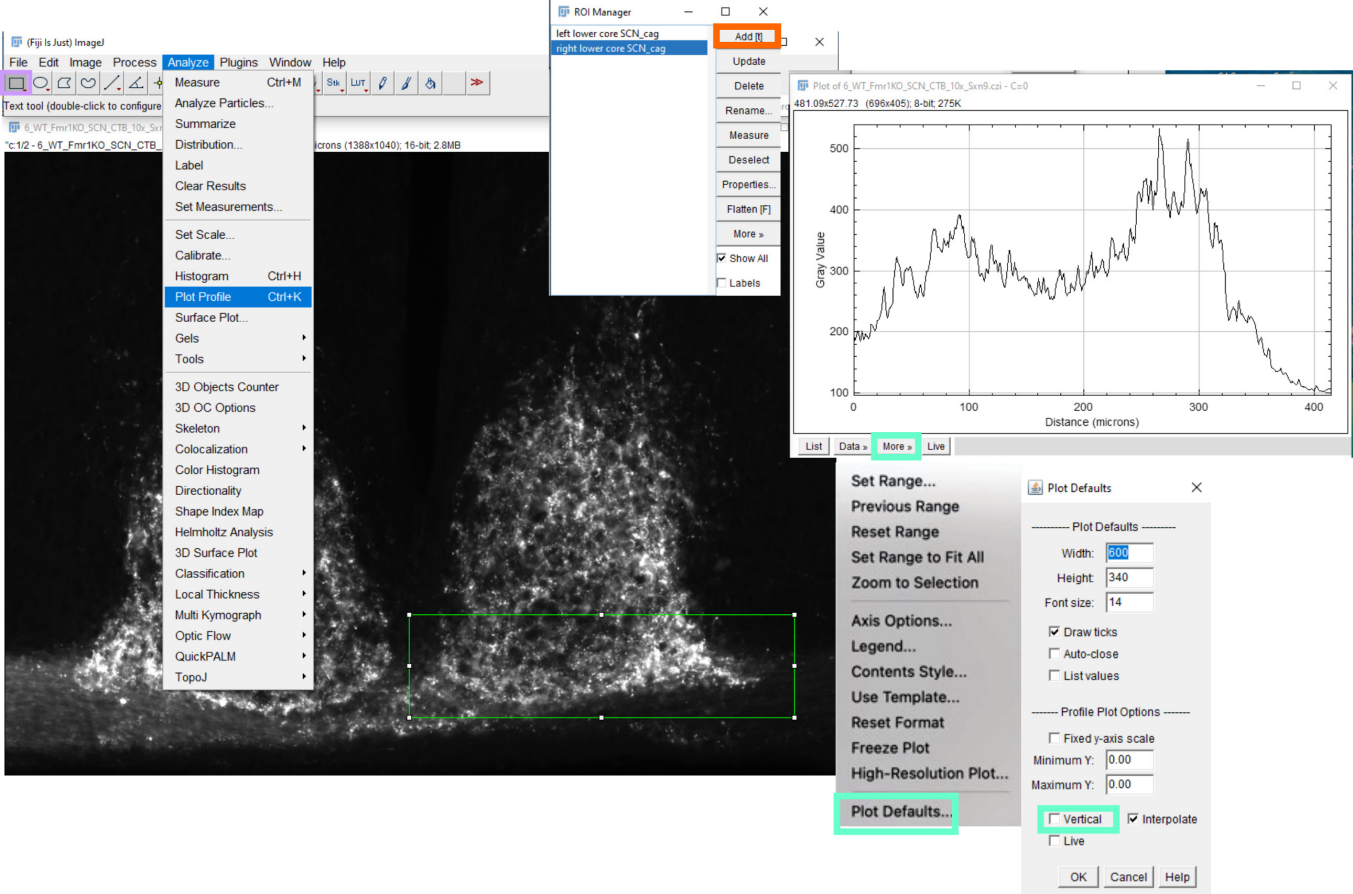
Using Fiji/ImageJ to detect deficits in the retino-hypothalamic pathway. Steps to set up the ROI using the *Rectangle* tool (Purple box) and obtain *Plot Profiles* of the distribution of the cholera toxin fluorescence intensity in the ventral suprachiasmatic nucleus (SCN). Orange box: click to *Add* the new ROI to the *ROI Manager*; Turquoise box: *More > Plot Defaults*, this pop-up window contains information on the size and type of plot.

2. Quantification of the fluorescent intensity signal distribution of the neuroanatomical tracer cholera toxin in the ventral SCN using the Profile Plot Plugin of Fiji/ImageJ:

a. Begin by following all the steps described in C1 from **a** to **e** (Figures 5, 6 and 7).

b. Select the *rectangle shape* (Figure 9, purple box) and set up the ROIs for the left and the right SCN using the cholera toxin images.

*Note: The* Plot Profile *only works with this shape.*

c. Draw a rectangular box to include the ventral part of either the left or right SCN and click *Add* in the ROI Manager (Figure 9, orange box). Save it directly from the ROI manager window (Figure 7, *More > save > location/folder*).

d. Carefully move the ROI to the other ventral SCN, position it properly, click *add* (Figure 9, orange box) in the ROI Manager window. The second ROI will be added. Save it.

*Notes:*

*1. Make sure that the shapes enclose at best all the afferent fibers in the ventral SCN. The ROIs might not be perfectly aligned in some of the images but a bit offset due to uneven mounting.*

*2. The ROI for the left and right SCN should be of the same fixed size (width × height).*

*3. The measures (width and height) of the ROIs can be found in* ROI manager *> More > Specify (Figure 7).*

e. To superimpose the ROI on the image: select the image and then the ROI in the window of the ROI manager; reposition it, **if** necessary, by using the arrows on the keyboard. Make sure not to change its size or shape! Click *Update* to save the change (Figure 7, magenta box) as needed; often, all the images for the same sample have the same alignment.

f. To generate a column plot profile: go to *ANALYZE > PLOT PROFILE* (or *Command/Control K*, Figure 9). A window with the plot of the intensity (gray value) will open (Figure 9).

g. The default is the horizontal plot; to change these settings, open an image, superimpose the ROI and obtain a plot profile.

h. In the menu at the bottom of the window of the plot, select *More* > *Plot Defaults* > *Vertical* (Figure 9, turquoise boxes). The new settings will remain unchanged for all the images to be assessed in that session. These should be checked at the beginning of each session.

**Figure 10.**
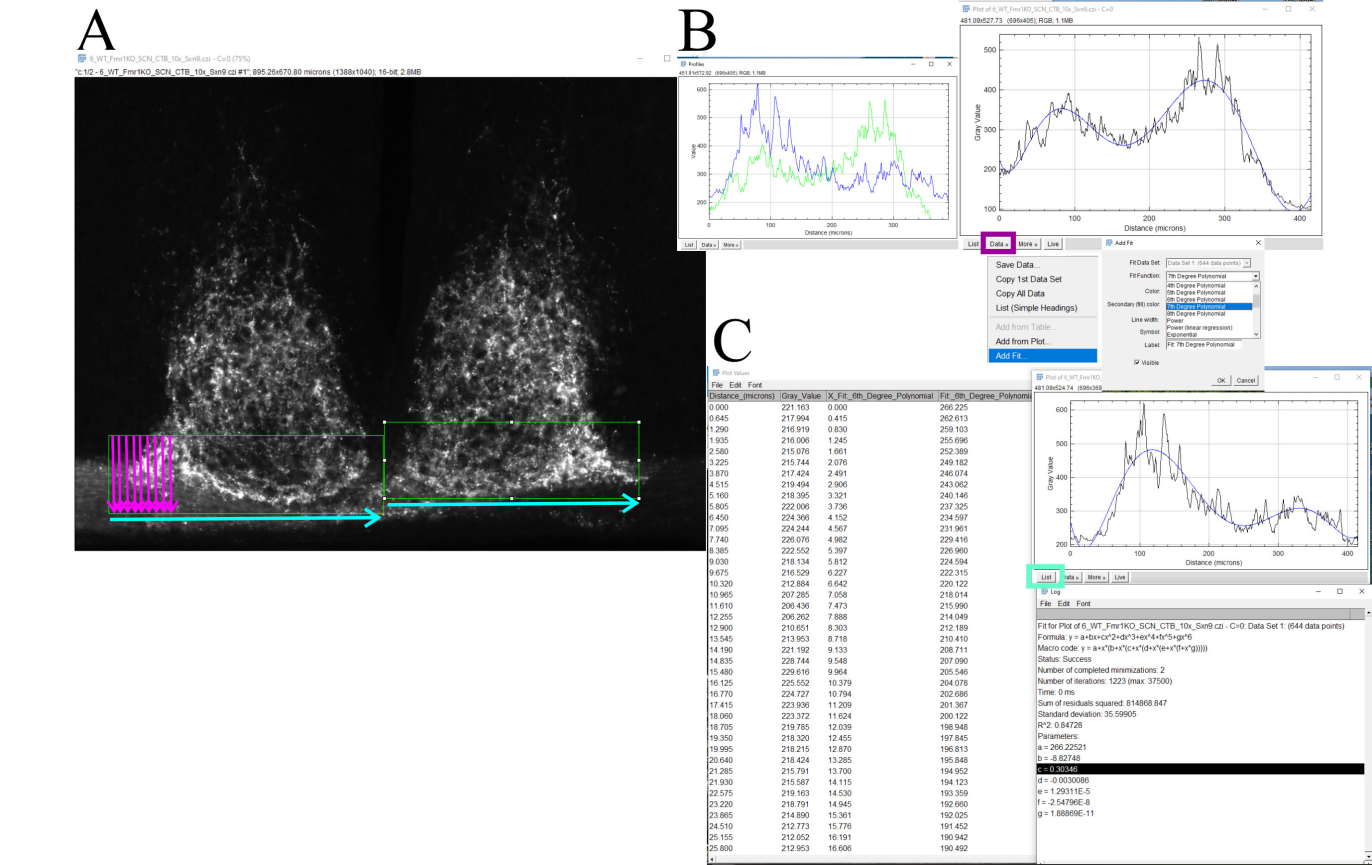
Using Fiji/ImageJ to detect deficits in the retino-hypothalamic pathway. (A) Schematic of the directionality for the measurement of the average intensity using the horizontal plot. The magenta arrows depict the direction of the densitometric scanning parallel to the short side (height) of the rectangle ROI and moving lengthwise (cyan arrows): lateral to medial for the left suprachiasmatic nucleus (SCN) and medial to lateral for the right SCN. (B) Examples of plots obtained for the left (blue trace) and right (green trace) SCN, in which the opposite position of the peaks of intensity for the cholera toxin signal in the most lateral parts of the nuclei can be appreciated. Deep violet box: *Data*, opens a menu with several functions to *save* the plots or add a *Fit function*. (C) Steps to extrapolate the values generated by the plots. Turquoise box: *List*; clicking on this will open the window with the numerical values of the plot.

*Note: In the horizontal plot, the average intensity is measured along the vertical y-axis or column. This can be described as a series of “lines or columns” that measures the staining intensity parallel to the height (Figure 10A, magenta arrows) of the rectangle moving lengthwise (lateral to medial for the left and medial to lateral for the right SCN; Figure 10A, cyan arrows); then, a column average plot is generated (Figures 9 and 10B). In this, the x-axis represents the horizontal distance through the SCN, whilst the y­axis represents the average pixel intensity per vertical line within the rectangular box (Figure 10B).*

i. Once the plots are obtained for several, if not for all, the images, before exporting the obtained values, in the *Plot* window, select *Data* (Figure 10B, deep violet box) > *Add Fit* to identify the polynomial that allows for the best estimation and alignment of the intensity peaks on the x-axis for all the left and right SCN.

j. Once the best fit is identified and applied to all plot profiles, save the data for each plot: click on *List* (Figure 10C, turquoise box) and a window with the values will open. To save all the values, go to *File > save as* (Figure 10C) or copy directly to an Excel spreadsheet.

We performed all the subsequent analyses of the plot profiles for the left and right SCN separately to obtain a single curve per side, per animal by averaging the profiles of the 5 sections. In our latest study [19], the fifth-order polynomial curves were found to be the best fit to estimate the position of the intensity peak on the x-axis; the original y-axis values were aligned using this position in Excel and averaged arithmetically to obtain one profile (either left or right).

## Data analysis

Data analyses are performed using Microsoft Excel and GraphPad Prism, as reported in [16,19]. The statistical tests to be used should be determined based upon the experimental design, the number of biological replicates/groups, and variables.

## Validation of protocol

This protocol (or part of it) was previously used in the following peer-reviewed research articles:

- Li et al. [14] Circadian rhythm disruption in a mouse model of Rett syndrome circadian disruption in RTT. *Neurobiology of Disease.* (Figure 3)
- Kuljis et al. [15] Sex Differences in Circadian Dysfunction in the BACHD Mouse Model of Huntington’s Disease. *PLoS ONE.* (Figure 3, Tables 5 and 6)
- Lee et al. [16] Sleep/Wake Disruption in a Mouse Model of BLOC-1 Deficiency. *Frontiers in Neuroscience.* (Figures 5 and 6)
- Wang et al. [19] Scheduled feeding improves behavioral outcomes and reduces inflammation in a mouse model of fragile X syndrome. *eLife.* (Figure 5, Table 4)
- Wang et al., [25] Melatonin treatment of repetitive behavioral deficits in the *Cntnap2* mouse model of autism spectrum disorder. *Neurobiology of Disease.* (Figure S7)
- Lee et al. [27] Sex-dimorphic effects of biogenesis of lysosome-related organelles complex-1 deficiency on mouse perinatal brain development. *Journal of Neuroscience Research.* (Table 3, Figures 2 and 3)

In [15], we reported sex differences in the size of the SCN in male and female wild-type mice, with the females having a smaller SCN (81%) than their male counterparts, as well as a significantly reduced SCN in male (87%), but not female, BACHD mice, a model of Huntington’s Disease, as compared to male wild types. *MeCp2*-null and BLOC-1-deficient male mice, two models of neurodevelopmental disabilities, displayed opposite phenotypes with, respectively a significantly smaller (14%) and larger (10%) SCN in comparison to their wild-type counterparts [14,16]. Based on our published results, we can infer that expected values for the area, height, and width of the mid-SCN in wild-type animals should be around 425,000–500,000 μm^2^, 300 μm, and 275 μm, respectively. Variability in the values obtained for the area (+/-100000) has been observed across the five studies; likely, these measurements may vary depending on the number of the sections containing the mid-SCN (5 vs. 7) used for the analysis, the type of staining (Nissl vs. DAPI staining) as well as the age of the animals.

## Supplementary information

The following supporting information can be downloaded here:

1. Supplementary methods

2. Supplementary Figure 1: Examples of outlined suprachiasmatic nuclei (SCN) in Nissl- (A) or DAPI- (B) stained sections.

## References

[1] Reppert S. M. and Weaver D. R. (2002). Coordination of circadian timing in mammals. Nature. 418(6901): 935 941 941. 10.1038/nature00965 12198538

[2] Moore R. Y., Speh J. C. and Leak R. K. (2002). Suprachiasmatic nucleus organization. Cell Tissue Res. 309(1): 89 98 98. 10.1007/s00441-002-0575-2 12111539

[3] Mohawk J. A., Green C. B. and Takahashi J. S. (2012). Central and Peripheral Circadian Clocks in Mammals. Annu Rev Neurosci. 35(1): 445 462 462. 10.1146/annurev-neuro-060909-153128 22483041 PMC3710582

[4] Colwell C. S. (2011). Linking neural activity and molecular oscillations in the SCN. Nat Rev Neurosci. 12(10): 553 569 569. 10.1038/nrn3086 21886186 PMC4356239

[5] Brown A. J., Pendergast J. S. and Yamazaki S. (2019). Peripheral Circadian Oscillators. Yale J Biol Med. 92(2): 327 335 335 . https://pubmed.ncbi.nlm.nih.gov/31249493/ 31249493 PMC6585520

[6] Milman N. E., Tinsley C. E., Raju R. M. and Lim M. M. (2023). Loss of sleep when it is needed most– Consequences of persistent developmental sleep disruption: A scoping review of rodent models. Neurobiol Sleep Circadian Rhythms. 14: 100085 10.1016/j.nbscr .2022.100085 36567958 PMC9768382

[7] Boivin D. B., Boudreau P. and Kosmadopoulos A. (2021). Disturbance of the Circadian System in Shift Work and Its Health Impact. J Biol Rhythms. 37(1): 3 28 28. 10.1177/07487304211064218 34969316 PMC8832572

[8] Colwell C. S. (2021). Defining circadian disruption in neurodegenerative disorders. J Clin Invest. 131(19): e1172/jci148288. 10.1172/jci148288 PMC8483739 34596047

[9] Bruni O., Breda M., Mammarella V., Mogavero M. P. and Ferri R. (2025). Sleep and circadian disturbances in children with neurodevelopmental disorders. Nat Rev Neurol. 21(2): 103 120 120. 10.1038/s41582-024-01052-9 39779841

[10] Wintler T., Schoch H., Frank M. G. and Peixoto L. (2020). Sleep, brain development, and autism spectrum disorders: Insights from animal models. J Neurosci Res. 98(6): 1137 1149 1149. 10.1002/jnr.24619 32215963 PMC7199437

[11] Medina E., Peterson S., Ford K., Singletary K. and Peixoto L. (2023). Critical periods and Autism Spectrum Disorders, a role for sleep. Neurobiol Sleep Circadian Rhythms. 14: 100088 10.1016/j.nbscr .2022.100088 36632570 PMC9826922

[12] Morton A. J. (2023). Sleep and Circadian Rhythm Dysfunction in Animal Models of Huntington’s Disease. J Huntington's Dis. 12(2): 133 148 148. 10.3233/jhd-230574 37334613 PMC10473141

[13] D. Dell’Angelica, Singh K., Colwell C. S. and Ghiani C. A. (2024). Circadian Interventions in Preclinical Models of Huntington’s Disease: A Narrative Review. Biomedicines. 12(8): 1777 10.3390/biomedicines12081777 39200241 PMC11351982

[14] Li Q., Loh D. H., Kudo T., Truong D., Derakhshesh M., Kaswan Z. M., Ghiani C. A., Tsoa R., Cheng Y., Sun Y. E., .(2015). Circadian rhythm disruption in a mouse model of Rett syndrome circadian disruption in RTT. Neurobiol Dis. 77: 155 164 164. 10.1016/j.nbd .2015.03.009 25779967

[15] Kuljis D. A., Gad L., Loh D. H., MacDowell Kaswan Z., Hitchcock O. N., Ghiani C. A. and Colwell C. S. (2016). Sex Differences in Circadian Dysfunction in the BACHD Mouse Model of Huntington’s Disease. PLoS One. 11(2): e0147583. 10.1371/journal.pone .0147583 PMC4752447 26871695

[16] Lee F. Y., Wang H. B., Hitchcock O. N., Loh D. H., Whittaker D. S., Kim Y. S., Aiken A., Kokikian C., Dell’Angelica E. C., Colwell C. S., .(2018). Sleep/Wake Disruption in a Mouse Model of BLOC-1 Deficiency. Front Neurosci. 12: e00759. 10.3389/fnins.2018 .00759 PMC6249416 30498428

[17] van Wamelen D. J., Aziz N. A., Anink J. J., van Steenhoven R., Angeloni D., Fraschini F., Jockers R., Roos R. A. C. and Swaab D. F. (2013). Suprachiasmatic Nucleus Neuropeptide Expression in Patients with Huntington's Disease. Sleep.: e2314. https://doi.org/10.5665/sleep.2314 PMC3524533 23288978

[18] La Morgia C., Ross‐Cisneros F. N., Koronyo Y., Hannibal J., Gallassi R., Cantalupo G., Sambati L., Pan B. X., Tozer K. R., Barboni P., .(2015). Melanopsin retinal ganglion cell loss in Alzheimer disease. Ann Neurol. 79(1): 90 109 109. 10.1002/ana.24548 26505992 PMC4737313

[19] Wang H. B., Smale N. E., Brown S. H., Villanueva S. A., Zhou D., Mulji A., Bhandal D. S., Nguyen-Ngo K., Harvey J. R., Ghiani C. A., .(2025). Scheduled feeding improves behavioral outcomes and reduces inflammation in a mouse model of fragile X syndrome. eLife. 14: e104720. https://doi.org/10.7554/elife.104720 PMC12422731 40928213

[20] Schmidt T. M., Do M. T. H., Dacey D., Lucas R., Hattar S. and Matynia A. (2011). Melanopsin-Positive Intrinsically Photosensitive Retinal Ganglion Cells: From Form to Function. J Neurosci. 31(45): 16094 16101 16101. 10.1523/jneurosci.4132-11 .2011 22072661 PMC3267581

[21] Schmidt T. M., Chen S. K. and Hattar S. (2011). Intrinsically photosensitive retinal ganglion cells: many subtypes, diverse functions. Trends Neurosci. 34(11): 572 580 580. 10.1016/j.tins .2011.07.001 21816493 PMC3200463

[22] Lazzerini Ospri L., Prusky G. and Hattar S. (2017). Mood, the Circadian System, and Melanopsin Retinal Ganglion Cells. Annu Rev Neurosci. 40(1): 539 556 556. 10.1146/annurev-neuro-072116-031324 28525301 PMC5654534

[23] Schroeder A. M. and Colwell C. S. (2013). How to fix a broken clock. Trends Pharmacol Sci. 34(11): 605 619 619. 10.1016/j.tips .2013.09.002 24120229 PMC3856231

[24] Valencia M. L., Sofela F. A., Jongens T. A. and Sehgal A. (2024). Do metabolic deficits contribute to sleep disruption in monogenic intellectual disability syndromes?. Trends Neurosci. 47(8): 583 592 592. 10.1016/j.tins .2024.06.006 39054162 PMC11997875

[25] Wang H. B., Tahara Y., Luk S. H. C., Kim Y. S., Hitchcock O. N., MacDowell Kaswan Z. A., In Kim Y., Block G. D., Ghiani C. A., Loh D. H., .(2020). Melatonin treatment of repetitive behavioral deficits in the Cntnap2 mouse model of autism spectrum disorder. Neurobiol Dis. 145: 105064 10.1016/j.nbd .2020.105064 32889171 PMC7597927

[26] Mullen B. R., Khialeeva E., Hoffman D. B., Ghiani C. A. and Carpenter E. M. (2012). Decreased Reelin Expression and Organophosphate Pesticide Exposure Alters Mouse Behaviour and Brain Morphology. ASN NEURO. 5(1): e1042/an20120060. 10.1042/an20120060 PMC3575035 23298182

[27] Lee F. Y., Larimore J., Faundez V., Dell’Angelica E. C. and Ghiani C. A. (2020). Sex‐dimorphic effects of biogenesis of lysosome‐related organelles complex‐1 deficiency on mouse perinatal brain development. J Neurosci Res. 99(1): 67 89 89. 10.1002/jnr.24620 32436302 PMC7677168

